# Celecoxib Adjunctive Treatment to Antipsychotics in Schizophrenia: A Review of Randomized Clinical Add-On Trials

**DOI:** 10.1155/2016/3476240

**Published:** 2016-07-25

**Authors:** Stefano Marini, Domenico De Berardis, Federica Vellante, Rita Santacroce, Laura Orsolini, Alessandro Valchera, Gabriella Girinelli, Alessandro Carano, Michele Fornaro, Francesco Gambi, Giovanni Martinotti, Massimo Di Giannantonio

**Affiliations:** ^1^Department of Neurosciences Clinical Imaging, Chair of Psychiatry, “G. d'Annunzio” University, 65100 Chieti, Italy; ^2^Polyedra Research Group, 64100 Teramo, Italy; ^3^NHS, Department of Mental Health, Psychiatric Service of Diagnosis and Treatment, Hospital “G. Mazzini”, ASL 4, 64100 Teramo, Italy; ^4^Villa S. Giuseppe Hospital, Hermanas Hospitalarias, 63100 Ascoli Piceno, Italy; ^5^School of Life and Medical Sciences, University of Hertfordshire, Hatfield AL10 9AB, UK; ^6^New York State Psychiatric Institute, Columbia University, New York, NY 10027, USA

## Abstract

Schizophrenia is a severe, chronic and debilitating mental disorder. Past literature has reported various hypotheses about the psychopathology of schizophrenia. Recently, a growing literature has been trying to explain the role of inflammation in the etiopathogenesis of schizophrenia. In the past, numerous immune modulation and anti-inflammatory treatment options have been proposed for schizophrenia, but sometimes the results were inconsistent. Electronic search was carried out in November 2015. PubMed and Scopus databases have been used to find studies to introduce in this review. Only randomized-placebo-controlled add-on trials were taken into account. In this way, six articles were obtained for the discussion. Celecoxib showed beneficial effects mostly in early stages of schizophrenia. In chronic schizophrenia, the data are controversial, possibly in part for methodological reasons.

## 1. Introduction

Schizophrenia is a severe, chronic, and debilitating mental disorder [1]. The adult prevalence is approximately 1%, but schizophrenics constitute close to 10% of the permanently disabled population [2].

Past literature has reported various hypotheses about the psychopathology of the schizophrenia. The dopamine hypothesis has tried to explain positive, negative, and cognitive symptoms of the schizophrenia suggesting different alterations of the dopamine activity in different brain regions [3–5]. The glutamate hypothesis suggested that phencyclidine and ketamine block of the N-methyl-D-aspartate receptor induced positive, negative, and cognitive symptoms [6–12]. The cytokine alterations in schizophrenics are consistent with the glutamate hypothesis of schizophrenia [13–17]. In fact, proinflammatory cytokines may influence dopaminergic and glutamatergic pathways and cognitive processes that are implicated in schizophrenia. Recently, alterations in the central gamma-aminobutyric acid and in the cholinergic systems have been proposed to be relevant for cognitive functions in schizophrenia [18–20].

A growing literature has been trying to explain the role of the inflammation in the pathophysiology of the schizophrenia. Emerging literature suggest that infectious exposures (e.g., influenza, genital reproductive infections,* Toxoplasma gondii,* and herpes simplex virus type 2) during the prenatal period may contribute to the etiopathogenesis of the schizophrenia [21–26]. Interesting data comes from animal studies of the maternal immune activation model of the schizophrenia. In animals, cytokines generated during the pregnancy may cross the placenta and blood-brain barrier and contribute to the oxidative stress [27–29].

A recent meta-analysis [30] has highlighted higher proinflammatory cytokines (IL-6, TNF-alpha, TGF-beta, and IFN-gamma) in acutely relapsed inpatients and in first-episode psychosis compared to controls. On the other hand, anti-inflammatory cytokine IL-10 levels were lower only in acutely relapsed patients with respect to controls.

The inflammatory hypothesis of the schizophrenia psychopathology has been supported by peripheral and central inflammatory signs in schizophrenic patients. Elevated proinflammatory factors, such as prostaglandin E2 (PGE2), C-reactive protein (CRP), interleukin- (IL-) 1beta, IL- 6, IL-8, and tumor necrosis factor- (TNF-) alpha, have been reported in serum/plasma levels (for recent reviews, see [31–33]).

Established correlations are that proinflammatory cytokines impair negative symptoms of schizophrenia [34, 35], deficiency in sustained attention [36], and psychomotor retardation [37]. Some authors have highlighted a positive correlation between the severity of cognitive deficit and increased levels of inflammatory markers in schizophrenic patients [38–40]. Proinflammatory cytokines have a role in altering the synthesis and the release of dopamine and noradrenalin [41–43]. This activity may play a role in the emergence of positive symptoms, but the studies have not found a significant correlation between positive symptoms and enhanced proinflammatory cytokines levels [44–46].

There are some evidences that antipsychotics may induce immune-modulatory effects [47–49]. Long-term treatment with antipsychotics exerts concomitant augmentation of anti-inflammatory cytokines (sIL-1RA, sIL-2R, and IL-10) [50–55] and a reduction of proinflammatory ones (IL-1beta, IL-6, sIL-6R, and TNF-alpha) [56–59]. Interestingly, second-generation antipsychotics may be more efficacious than first generations in enhancing anti-inflammatory cytokines (reviewed in [60, 61]). Moreover, some authors have reported that, in drug resistant schizophrenia patients, immune abnormalities cannot be normalized [62].

Past literatures have reported decreases in volume of the central nervous system in schizophrenia, already during the first episode, especially in schizophrenics with a poor outcome [63, 64]. Moreover, some authors have showed a relationship between brain volume, IL-1, and IL-6 [65, 66]. Numerous imaging studies have reported microglia activation and alteration in astrocytes population in the brains of recent onset and chronic schizophrenics, supporting the neuroinflammation hypothesis [67–69]. Some small postmortem studies have indicated an increased immunoreactive microglia in schizophrenic patients [70–72].

The tryptophan metabolism has been hypothesized to have a role in the etiopathogenesis of the schizophrenia. In fact, increased levels of kynurenic acid have been found in particular brain regions of schizophrenics [73–75]. Moreover antipsychotic treatments have an impact on kynurenic acid levels in humans and rats [76].

In the past, numerous immune modulation and anti-inflammatory treatment options have been proposed for schizophrenia, but sometimes the results were inconsistent. Between the immune modulation options, authors proposed omega-3 fatty acids [77–81], erythropoietin [82, 83], tetracycline antibiotic [84], minocycline [85–89], azithromycin [90], and valacyclovir [91]. Between the anti-inflammatory options, some trials were conducted with acetylsalicylic acid [92, 93], neurosteroids, and pregnenolone [94–96]. Recently, N-acetyl-cysteine add-on to anti-inflammatory agents has been tried [97, 98]. Emerging and promising adjunctive treatments for schizophrenia are represented by hormones (e.g., estrogen and oxytocin) [99–101], glutamatergic (e.g., glycine and d-serine) [102, 103], and nicotinergic compounds (e.g., varenicline and galantamine) [104, 105] and cannabidiol [106] (for an extensive review, see [107]).

## 2. Materials and Methods

Electronic search was carried out in November 2015. PubMed and Scopus databases have been used to find studies to introduce in this review. Keywords used in the search process were represented by “celecoxib add-on to antipsychotics”, “celecoxib adjunctive treatment to antipsychotics”, “celecoxib treatment for schizophrenia”, and “celecoxib treatment for schizophrenics”. Moreover, also studies found by hand search have been obtained to be included in this paper. Only randomized-placebo-controlled add-on trials were taken into account. In this way, six articles were obtained for the discussion.

## 3. Discussion

In literature only six randomized-placebo-controlled celecoxib add-on to antipsychotics trials are present [108–113] (see Table 1).

There are two variants of cyclooxygenase (COX) enzyme: COX-1 and COX-2. Celecoxib is a selective inhibitor of COX-2. Both variants function in the promotion of inflammation, pain, and fever, but only COX-2 plays an important role in the central nervous system [115]. Contrary to COX-1 inhibitors which could cause psychotic symptoms and cognitive dysfunctions, the therapeutic effect of celecoxib in schizophrenia is represented by the COX-2 inhibitor-mediated decrease of kynurenine levels [116].

Effects of celecoxib have been studied in add-on to risperidone, to olanzapine in one study, and to amisulpride in another. Only one study used constant doses of antipsychotics [110], the others used flexible doses. In each trial, 400 mg/day of celecoxib was administered. Durations of trials were different between studies, except for two. Even if the subjects enrolled in the studies were all schizophrenics, some differences have to be highlighted. In fact, two studies enrolled first manifestation schizophrenics [111, 113], whilst the others recruited continuously ill or chronic in active phase patients.

Four studies showed at least partial benefit in the celecoxib add-on treatment to antipsychotics groups based on the Positive and Negative Syndrome Scale (PANSS) [117] or Clinical Global Impressions scale (CGI) [118]. Even if two studies were conducted in particular patient populations (Chinese first manifestation and Iranian chronic schizophrenics) [111, 112], the adjunctive celecoxib treatment reported similar benefits than other populations.

Since previous trials had been performed with risperidone, Müller et al. [113] decided to add celecoxib to amisulpride. A significantly better outcome in both positive and negative symptoms was observed in the group treated with adjunctive celecoxib compared to the placebo group. For the first time, a pronounced effect by celecoxib on schizophrenic negative symptoms was demonstrated [113], even if the particular effect of amisulpride on these symptoms is well known [119].

Two studies have not shown any therapeutic effect [109, 110], maybe due to the patient selection (refractory schizophrenia) and the different antipsychotics utilized (risperidone and olanzapine) and the duration of the trials. In fact, in animal studies observed that the effects of celecoxib on cytokines and behavioural symptoms depend on the time of administration of celecoxib [120]. Moreover, since first episodes of schizophrenia are easier to treat than recurrent manifestation [121], these negative reports may have been due to chronic conditions.

A recent and useful meta-analysis [122] has investigated five randomized clinical trials reporting data on 264 patients in treatment with Non-Steroid Anti-Inflammatory Drugs (NSAID) augmentation to antipsychotics in schizophrenia. The authors calculated the effect of NSAIDs on symptom severity measured with the Positive and Negative Syndrome Scale (PANSS) [117]. Likewise to our review, NSAID adjunctive treatment to antipsychotics showed a moderate beneficial effect on total symptom severity as well as on positive symptoms in schizophrenia and a small effect on negative symptoms in schizophrenia.

Very recently, Baheti et al. [123] have conducted an open-labeled, prospective, 6-week controlled trial to evaluate the effect of celecoxib as add-on to olanzapine therapy in acute exacerbation schizophrenics. Beneficial effects in positive, negative, and general psychopathology and total scores on PANSS have been reported, maybe for the short-term period of the trial and for the use of ICD-10 criteria for the diagnosis of schizophrenia.

Side effects were not significantly different between groups. Two trials used biperiden and lorazepam to treat extrapyramidal and anxiety side effects [108, 112]. Drop-out rates were different between trials. Even if the number of patients who dropped out from the trial was low, data are contrasting (major in celecoxib group [108], major in placebo group [112, 113]). Other studies have not reported drop-out rates [109–111].

Since inflammation has been correlated with the development of insulin resistance and metabolic disturbances [124], which are frequent in schizophrenics increased also by the antipsychotics [31], the use of anti-inflammatory agents may be very useful in future treatments.

In recent years, innovative therapies for autoimmune diseases have provided futuristic candidate agents for the cytokine based treatment of the schizophrenia. These treatment are based on antibodies or antibody components against cytokines or cytokine receptors [125–127].

## 4. Conclusions

A growing literature has been trying to explain the role of the inflammatory process in the pathophysiology of the schizophrenia. In the past, numerous studies have proposed anti-inflammatory treatment for schizophrenics, but sometimes results were inconsistent. Recently some trials have studied the effects of celecoxib add-on to risperidone, to olanzapine, and to amisulpride. Celecoxib showed beneficial effects mostly in early stages of the schizophrenia. In chronic schizophrenics the data are controversial, possibly in part for methodological reasons. In the authors' opinion, future research should investigate celecoxib alone in the treatment of schizophrenia symptoms to better evaluate the schizophrenia inflammatory hypothesis and the real effect of the COX-2 inhibitor.

## Figures and Tables

**Table 1:** Celecoxib randomized clinical add-on trials.

Authors	Study design	Participants	Treatment duration	Celecoxib doses	Antipsychotics	Major findings
Müller et al. 2002 [108]	Double-blind, randomized, placebo-controlled, add-on	*N* = 50 Schizophrenics Duration of illness not specified (mean 5.9 years)	5 weeks	400 mg/day	Risperidone (flexible dose)	Significant advantage of the COX-2 inhibitor

Rappart and Müller 2004 [109]	Double-blind, randomized, placebo-controlled, add-on	*N* = 270 Schizophrenics Duration of illness ≤10 years	11 weeks	400 mg/day	Risperidone (flexible dose)	No advantage on the COX-2 inhibitor

Rapaport et al. 2005 [110]	Double-blind, randomized, placebo-controlled, add-on	*N* = 38 Schizophrenics Continuously ill (mean 20 years)	8 weeks	400 mg/day	Risperidone or olanzapine (constant dose)	No advantage on the COX-2 inhibitor

Zhang et al. 2006 [111]	Double-blind, randomized, placebo-controlled, add-on	*N* = 40 First manifestation schizophrenia	12 weeks	400 mg/day	Risperidone (flexible dose)	Significant advantage of the COX-2 inhibitor

Akhondzadeh et al. 2007 [112]	Double-blind, randomized, placebo-controlled, add-on	*N* = 60 Active phase of chronic schizophrenia	8 weeks	400 mg/day	Risperidone (flexible dose)	Significant advantage of the COX-2 inhibitor

Müller et al. 2010 [113]	Double-blind, randomized, placebo-controlled, add-on	*N* = 49 First manifestation schizophrenia	6 weeks	400 mg/day	Amisulpride (flexible dose)	Significant advantage of the COX-2 inhibitor

Adapted from [114].
